# Updating the approaches to define susceptibility and resistance to anti-tuberculosis agents: implications for diagnosis and treatment

**DOI:** 10.1183/13993003.00166-2022

**Published:** 2022-04-14

**Authors:** Antimycobacterial Susceptibility Testing Group, Sophia B. Georghiou, Timothy C. Rodwell, Alexei Korobitsyn, Said H. Abbadi, Kanchan Ajbani, Jan-Willem Alffenaar, David Alland, Nataly Alvarez, Sönke; Andres, Elisa Ardizzoni, Alexandra Aubry, Rossella Baldan, Marie Ballif, Ivan Barilar, Erik C. Böttger, Soumitesh Chakravorty, Pauline M. Claxton, Daniela M. Cirillo, Iñaki Comas, Chris Coulter, Claudia M. Denkinger, Brigitta Derendinger, Edward P. Desmond, Jurriaan E.M. de Steenwinkel, Keertan Dheda, Andreas H. Diacon, David L. Dolinger, Kelly E. Dooley, Matthias Egger, Soudeh Ehsani, Maha R. Farhat, Lanfranco Fattorini, Iris Finci, Laure Fournier Le Ray, Victoria Furió, Ramona Groenheit, Tawanda Gumbo, Scott K. Heysell, Doris Hillemann, Harald Hoffmann, Po-Ren Hsueh, Yi Hu, Hairong Huang, Alamdar Hussain, Farzana Ismail, Kiyohiko Izumi, Tomasz Jagielski, John L. Johnson, Priti Kambli, Koné Kaniga, G.H.R. Eranga Karunaratne, Meenu Kaushal Sharma, Peter M. Keller, Ellis C. Kelly, Margarita Kholina, Mikashmi Kohli, Katharina Kranzer, Ian F. Laurenson, Jason Limberis, S-Y. Grace Lin, Yongge Liu, Alexandre López-Gavín, Anna Lyander, Diana Machado, Elena Martinez, Faisal Masood, Satoshi Mitarai, Nomonde R. Mvelase, Stefan Niemann, Vladyslav Nikolayevskyy, Florian P. Maurer, Matthias Merker, Paolo Miotto, Shaheed V. Omar, Ralf Otto-Knapp, Moisés Palaci, José Juan Palacios Gutiérrez, Sharon J. Peacock, Charles A. Peloquin, Jennifer Perera, Catherine Pierre-Audigier, Suporn Pholwat, James E. Posey, Therdsak Prammananan, Leen Rigouts, Jaime Robledo, Neesha Rockwood, Camilla Rodrigues, Max Salfinger, Marcos C. Schechter, Marva Seifert, Sarah Sengstake, Thomas Shinnick, Natalia Shubladze, Vitali Sintchenko, Frederick Sirgel, Sulochana Somasundaram, Timothy R. Sterling, Andrea Spitaleri, Elizabeth Streicher, Philip Supply, Erik Svensson, Elisa Tagliani, Sabira Tahseen, Akiko Takaki, Grant Theron, Gabriela Torrea, Armand Van Deun, Jakko van Ingen, Annelies Van Rie, Dick van Soolingen, Roger Vargas Jr, Amour Venter, Nicolas Veziris, Cristina Villellas, Miguel Viveiros, Robin Warren, Shu'an Wen, Jim Werngren, Robert J. Wilkinson, Caie Yang, F. Ferda Yılmaz, Tingting Zhang, Danila Zimenkov, Nazir Ismail, Claudio U. Köser, Thomas Schön

**Affiliations:** Collaborators are listed at the end of the article

## Abstract

Approximately 85 000 deaths globally in 2019 were due to drug-resistant tuberculosis (TB), which corresponds to 7% of global deaths attributable to bacterial antimicrobial resistance [1]. Yet concerns have been mounting that drug-resistant TB was being underestimated because the approaches to define susceptibility and resistance to anti-TB agents had not kept up with those used for other major bacterial pathogens [2–9]. Here, we outline the recent, evidence-based initiatives spearheaded by the World Health Organization (WHO) and others to update breakpoints (traditionally referred to as critical concentrations (CCs)) that are used for phenotypic antimicrobial susceptibility testing (AST), also called drug susceptibility testing in the TB literature.

## Abstract 2

**Inappropriately high breakpoints have resulted in systematic false-susceptible AST results to anti-TB drugs. MIC, PK/PD and clinical outcome data should be combined when setting breakpoints to minimise the emergence and spread of antimicrobial resistance.** https://bit.ly/3i43wb6

Approximately 85 000 deaths globally in 2019 were due to drug-resistant tuberculosis (TB), which corresponds to 7% of global deaths attributable to bacterial antimicrobial resistance [1]. Yet concerns have been mounting that drug-resistant TB was being underestimated because the approaches to define susceptibility and resistance to anti-TB agents had not kept up with those used for other major bacterial pathogens [2–9]. Here, we outline the recent, evidence-based initiatives spearheaded by the World Health Organization (WHO) and others to update breakpoints (traditionally referred to as critical concentrations (CCs)) that are used for phenotypic antimicrobial susceptibility testing (AST), also called drug susceptibility testing in the TB literature.

WHO commissioned five reports that considered studies in up to 16 languages from a wide diversity of global contributors to ensure that the compiled data were as comprehensive as possible. The first report consisted of a systematic review that covered publications relating to the CCs of the most important drugs for the treatment of multidrug-resistant (MDR) or rifampicin-resistant (RR) TB, including newly approved bedaquiline and delamanid [10]. The second report was an accompanying background document on the pharmacokinetics and pharmacodynamics (PK/PD) of those drugs, whereas the third presented the findings of a meta-analysis of clinical outcome data [11, 12]. The fourth was a systematic review of the CCs for the rifamycins and isoniazid [13]. Finally, WHO released its first official catalogue of resistance mutations to inform the interpretation of genotypic AST results [14, 15]. Together, these reports prompted WHO to make major changes to its recommendations for TB treatment (*e.g.* kanamycin is no longer recommended for the treatment of TB (figure 1b)) and AST, as discussed below [16].

**FIGURE 1:**
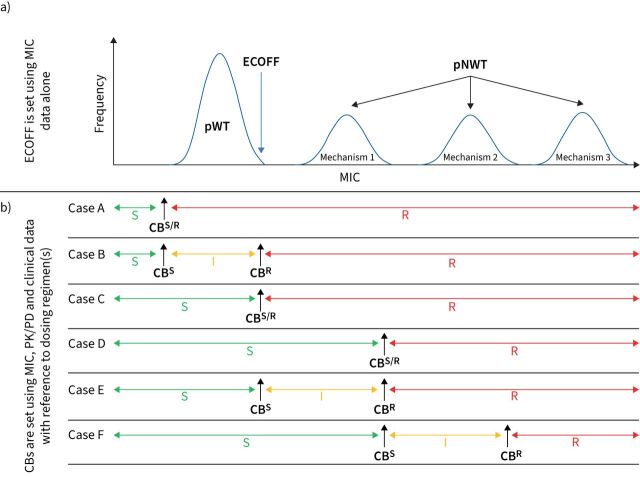
The European Committee on Antimicrobial Susceptibility Testing (EUCAST) approach for setting breakpoints compared with the World Health Organization (WHO). a) Four hypothetical minimum inhibitory concentration (MIC) distributions of an antibiotic for the same species. The distribution with the lowest MICs is typically the phenotypically wild-type (pWT) distribution, whereas the remaining three are phenotypically non-wild type (pNWT) with different underlying mechanisms. Notably, the upper end of the pWT distribution, which corresponds to the epidemiological cut-off value (ECOFF), does not automatically become a clinical breakpoint (CB), as shown in panel (b). Instead, pharmacokinetic/pharmacodynamic (PK/PD) and clinical data must be analysed to assess whether any of the represented populations are susceptible (S), susceptible at increased exposure (I), or resistant (R) [17, 24]. This may demonstrate that an agent offers no clinical benefits even for pWT strains at clinically attainable drug exposures, in which case the species in question would be deemed to be intrinsically resistant (case A). In 2018, WHO reached this conclusion for kanamycin and capreomycin after decades of clinical use globally, which prompted their withdrawal from clinical recommendations, although the underlying meta-analysis has attracted criticism [12, 16, 33, 34]. If a drug is clinically effective, one of five scenarios may apply. First, the pWT population may only be susceptible at increased exposure (case B). This uncommon approach is used to minimise the chance of clinicians prescribing the wrong regimen if a lower dose is commonly used for other pathogens. Second, the standard dosing regimen of the drug may be sufficient to treat only the pWT population (case C). This is the most common scenario when a drug is first approved and there is clinical outcome data to support its efficacy for the pWT population, whereas sufficient PK/PD and extensive clinical data in support of higher doses or treatment of pNWT isolates with resistance mechanisms are usually lacking. Gathering sufficient clinical outcome data for different pNWT populations is particularly challenging for TB given that multidrug regimens are always used, which may result in synergies or antagonism between one or more agents [27]. Nevertheless, the impact of individual mutations can be correlated with clinical outcomes, particularly for core drugs, provided that the studies are sufficiently powered [19, 25, 35, 36]. Third, the standard dosing regimen may also be sufficiently potent to treat strains with mechanism 1 but not strains with higher MICs because of mechanisms 2 and 3 (case D). Fourth, mechanism 1 may only be treatable at an increased exposure, as shown in case E. Finally, case F represents a hybrid between cases D and E. The current WHO definition of the critical concentration (CC) is effectively that of an ECOFF (*i.e.* it is set based on MIC data alone, taking genotypic information into consideration when relevant) even though the CC is actually used as a CB^S/R^ (*i.e.* pWT strains are reported as susceptible and pNWT strains as resistant based on a limited review of clinical evidence and PK/PD data compared with other bacterial pathogens) [10, 12, 13, 16]. The only exception is moxifloxacin (table 1), for which the CC is used as a CB^S^ and the CB^WHO^, as defined by WHO, is effectively a CB^R^ (case E), which may cause confusion with some clinicians who rarely treat TB. Moreover, this contradicts the assertion that an “intermediate” category, which is an alternative term to describe MIC increases that can be overcome by dose increases, does not exist for TB [10, 13, 24].

For most antimicrobials with proven clinical efficacy at a specific dose, only the phenotypically wild-type (pWT) population of the bacterium in question is considered treatable (figure 1a) [17]. Thus, the main aim of the two systematic reviews commissioned by WHO was to evaluate the available minimum inhibitory concentration (MIC) data to assess whether existing CCs corresponded to epidemiological cut-off values (ECOFFs), which represent the upper end of the pWT MIC distribution (figure 1a) [10, 13]. This revealed limitations in both the quality and quantity of available MIC data in the TB field, in contrast to many other major bacterial pathogens [8, 9]. In fact, the data for most drug-medium combinations did not meet the criteria set out by the European Committee on Antimicrobial Susceptibility Testing (EUCAST) for setting ECOFFs [7, 9]. Faced with this situation and the critical, global need for AST guidance, WHO adopted a pragmatic approach and set 12 new CCs for second-line drugs based on systematic reviews of available existing data, while clearly highlighting that these decisions should be re-evaluated once additional data become available [7, 10]. Even using these less stringent criteria, three previously endorsed CCs had to be withdrawn owing to a clear lack of supporting evidence. This included the only CC for cycloserine available up to that point, which means that phenotypic AST is not possible and, consequently, patients with resistant strains are needlessly at risk of the sometimes severe side-effects of this drug [10, 16].

More importantly, two previous CCs for rifampicin, arguably amongst the most significant breakpoints in diagnostic microbiology, were found to be too high, leading to false-susceptible AST results for some isolates (*i.e.* very major diagnostic errors that increase the likelihood of treatment failure and selection of resistance to other drugs) [13, 18, 19]. In fact, the rifampicin CC for Middlebrook 7H10 medium was twice as high as the ECOFF for more than half a century [18]. The therapeutic impact of these diagnostic misclassifications was that some phenotypically non-wild type isolates were deemed treatable with the recommended 10 mg·kg^−1^ body weight per day dose despite a lack of PK/PD or clinical evidence (case D in figure 1b) [13, 18]. WHO, consequently, lowered the rifampicin CCs to the tentative ECOFFs based on data from the systematic review (case C in figure 1b) [13]. To minimise false-susceptible results, the WHO-endorsed CCs of three second-line drugs (amikacin, levofloxacin and moxifloxacin) were also lowered to their respective ECOFFs [10]. In the case of moxifloxacin, approximately 90% of resistant isolates could have been misclassified as susceptible using only the WHO CC of 2 mg·L^−1^ for BACTEC Mycobacterial Growth Indicator Tube system by Becton Dickinson that was valid between 2014 and 2018 [20]. Fortunately, a number of practical factors (table 1) meant that the clinical consequences of this incorrectly set CC were reduced considerably.

**TABLE 1:** Overview of changes to moxifloxacin breakpoints

**Medium**		**Recommended moxifloxacin breakpoint (in mg·L** ^−1^ **); daily dose (in mg)** ^¶^			
		**CLSI**	**WHO**		
		**Since 2011 [37, 43, 44]**	**2008–2014 [45, 46]**	**2014–2018 [47]**	**Since 2018/19 [10, 48]**
7H10	CC	0.5^+^; N/A^§^	−	**0.5**; N/A^*f*^ **2**; **400**^##^	**0.5**^¶¶^; **400 (standard dose)** in longer regimen or **400–800 (high dose)** in shorter regimen^++^
	CB^WHO^	−	−	−	**2**; **400–800 (high dose)** in longer regimen^++^
MGIT	CC	0.25^+^; N/A^§^	0.25; 400	**0.5**; N/A^*f*^ **2**; **400**^##^	**0.25**^¶¶^; **400 (standard dose)** in longer regimen or **400–800 (high dose)** in shorter regimen^++^
	CB^WHO^	−	−	−	**1**; **400–800 (high dose)** in longer regimen^++^

Based on World Health Organization (WHO) surveillance data^#^, approximately 90% of moxifloxacin-resistant isolates could have been misclassified as susceptible using the BACTEC Mycobacterial Growth Indicator Tube (MGIT) because the WHO critical concentration (CC) of 2 mg·L^−1^ was eight times higher than the epidemiological cut-off value (ECOFF) between 2014 and 2018. In practice, however, the rate of misclassification was far lower. First, many countries did not use moxifloxacin at all during this period and, consequently, did not use this CC. Second, even countries that prescribed moxifloxacin avoided or minimised the misclassifications because they completely or primarily relied on genotypic antimicrobial susceptibility testing, continued using the MGIT CC of 0.25 mg·L^−1^ in accordance with the Clinical and Laboratory Standards Institute (CLSI) guidelines, used a CC for another fluoroquinolone as surrogate for moxifloxacin resistance, or relied on 0.5 mg·L^−1^ as the breakpoint for the standard dose of moxifloxacin in combination with 2 mg·L^−1^ as the breakpoint for the high dose of moxifloxacin. The theoretical rate of false-susceptible results was lower using Middlebrook 7H10 medium because the WHO CC of 2 mg·L^−1^ was four rather than eight times higher than the ECOFF. The clinical breakpoints introduced by WHO (CB^WHO^) are not recognised by CLSI. ^#^: comparing MGIT results for 2 mg·L^−1^ moxifloxacin with 2 mg·L^−1^ ofloxacin in the WHO surveillance study, which is equivalent to testing the currently recognised levofloxacin CC of 1 mg·L^−1^ (*i.e.* 1.5 mg·L^−1^ tested in that study was also too high) [10, 20]. ^¶^: changes to WHO breakpoint/dose combinations relative to the previous guidelines are highlighted in bold. Breakpoints that correspond to ECOFFs are underlined [10]. ^+^: can be tested as surrogate for other fluoroquinolones [37, 43–45]. ^§^: not applicable (N/A) as CLSI does not define doses for treatment. ^*f*^: 0.5 mg·L^−1^ moxifloxacin in 7H10 and MGIT were recommended as surrogates for resistance to ofloxacin and levofloxacin. Because the ECOFF for moxifloxacin is 0.25 mg·L^−1^ in MGIT, this meant that some strains resistant to ofloxacin and levofloxacin were misclassified as susceptible [10]. In effect, the surrogate breakpoints at 0.5 mg·L^−1^ and moxifloxacin CCs at 2 mg·L^−1^ were set inconsistently for both media because the 7H10 data was extrapolated to MGIT, despite the systematic differences between both media [47]. ^##^: the WHO-endorsed dosage for individualised multidrug-resistant/rifampicin-resistant tuberculosis (MDR/RR-TB) regimens was 400 mg [47]. However, operational research using a higher dosage of moxifloxacin (800 mg) in a standardised short-course MDR/RR-TB regimen was in progress, although not WHO-endorsed at the time [49]. ^¶¶^: not recommended as surrogate for other fluoroquinolones [10]. ^++^: levofloxacin is the preferred fluoroquinolone for the shorter all-oral bedaquiline-containing MDR/RR-TB regimen recommended by WHO in 2020, but high-dose moxifloxacin can be used instead. However, any moxifloxacin resistance, irrespective of the level, is an exclusion criterion for the shorter all-oral regimen (*i.e.* the CC is the relevant breakpoint) [48]. This exclusion criterion for moxifloxacin had also applied to the shorter amikacin-containing MDR/RR-TB regimen that was recommended by WHO between 2018 and 2020 [50]. High-dose moxifloxacin can only be used to treat low-level resistant strains as part of the longer MDR/RR-TB regimen, for which the CB^WHO^ is valid [48].

When CCs are too high, they may not only result in undertreatment of the patient based on phenotypic AST, but can also adversely affect the design and interpretation of genotypic AST methods that represent the most viable option to scale up AST globally [18, 19, 21]. Between 2011 and 2014, for instance, the WHO-endorsed GenoType MTBDR*plus* VER 2.0 by Hain Lifescience was designed not to detect *rpoB* L452P because this mutation was not considered to be a rifampicin resistance mutation at that time [18, 19]. It took more than a decade for *eis* c-14t and *rrs* c1402t to be recognised as resistance mutations for amikacin [14, 15, 22]. Hence, the full potential of the GenoType MTBDR*sl* VER 2.0 was not exploited because these two mutations were only interpreted as markers for kanamycin and capreomycin resistance.

Another consequence of CCs that are too high is an unnecessarily high number of clinical isolates that are genotypically resistant (*i.e.* contain mutations associated with resistance) but test phenotypically susceptible. This has resulted in underestimates of the accuracy of genotypic methods when phenotypic AST has been used as a reference and, consequently, reduced the confidence in genotypic AST. This apparent discordance also obscured the fact that clinically relevant mutations for some drugs cannot be reliably confirmed by current phenotypic AST methods, even if the CC corresponds to the ECOFF, because the MIC distributions of susceptible and resistant strains overlap based on current data (figure 2) [23]. This is the case for rifampicin and, therefore, WHO has adopted a composite reference standard to ensure that borderline *rpoB* resistance mutations are not missed (*i.e.* an isolate is now considered resistant to rifampicin if it tests resistant by phenotypic AST or harbours a recognised resistance mutation, provided that the pre-test probability is considered) [13, 18, 19, 23]. However, clear and user-friendly guidance on how to resolve discordances during routine clinical care is also needed for other drugs [23].

**FIGURE 2:**
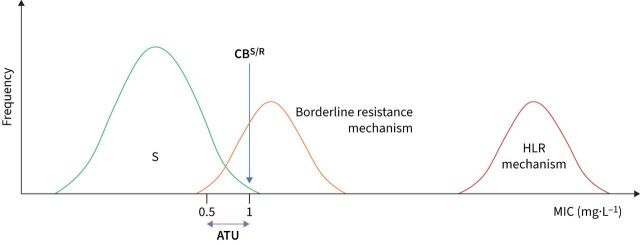
Strategies to minimise false-susceptible results by phenotypic antimicrobial susceptibility testing (AST) linked to borderline resistance mechanisms. Unlike the idealised scenario depicted in figure 1a, borderline resistance mechanisms exist with minimum inhibitory concentration (MIC) distributions that overlap with the susceptible distribution (*e.g.* the seven borderline rifampicin resistance mutations in *rpoB*) [10, 13, 37–39]. A clinical breakpoint (CB^S/R^) that corresponds to the epidemiological cut-off value (ECOFF) (case C in figure 1b) intersects the MIC distributions of such mechanisms (at 1 mg·L^−1^ in the hypothetical example below). Even if such an isolate is tested multiple times in the same laboratory, it will variably test susceptible and resistant because of the inherent technical variability of phenotypic AST [18]. Four measures that are not mutually exclusive can be taken to decrease such false-susceptible results. First, the optimal solution would be to eliminate or at least minimise the degree of overlap between distributions by reducing the technical variability of MIC testing as much as possible, which was one of the reasons that prompted the European Committee on Antimicrobial Susceptibility Testing (EUCAST) to develop its reference method and associated procedures to improve quality control [8, 9, 40, 41]. Second, EUCAST has introduced areas of technical uncertainty (ATUs) [24]. In this example, an MIC result of ≤0.5 mg·L^−1^ would be reported as susceptible, whereas MICs of >1 mg·L^−1^ would be resistant. By contrast, an MIC result of 1 mg·L^−1^ would be “uncertain” as the isolate in question could not be unequivocally classified as either susceptible or resistant based on the single MIC result because of the overlapping MIC distributions (*i.e.* this applies to the borderline resistance mechanism but not high-level resistance (HLR) mechanism) [18]. Although the prevalence of borderline resistance in a particular setting can give an indication of which of these possibilities is more likely, other experimental results are needed to resolve this situation conclusively. For example, if the molecular basis of the borderline resistance mechanism is known and is detected, the isolate could be reported as resistant (*i.e.* a composite reference standard is used, as WHO recommends for rifampicin) [18, 19, 23]. In fact, the Clinical and Laboratory Standards Institute (CLSI) has set an “inconclusive” category for ethambutol for the Sensititre MYCOTB plate by Thermo Fisher Scientific, which appears to serve as an ATU to minimise false susceptibility due to *embB* mutations [37, 39]. Third, adopting interpretative reading, whereby the results of two antibiotics that share at least one resistance mechanism are analysed together, may be useful (*e.g.* if the MICs for bedaquiline and clofazimine are equal to or just above the CB^S/R^, it is likely that the isolate in question has an *Rv0678* mutation) [38]. Finally, a surrogate agent could be tested that provides a better resolution between the relevant distribution (*e.g.* CLSI and EUCAST recommend pefloxacin as a surrogate for fluoroquinolone resistance in *Salmonella enterica*) [42].

More fundamentally, MIC, PK/PD and clinical data should be fully integrated when setting breakpoints [11, 17]. In 2018, WHO endorsed a second breakpoint for moxifloxacin that is higher than the CC in support of high-dose moxifloxacin treatment as part of the longer individualised MDR/RR-TB regimen (table 1) [10]. A “susceptible at increased exposure” range thus was defined, though this specific terminology was not used in the report (case E in figure 1b) [24]. The primary justification for this decision relied on extrapolating clinical outcome data for high-dose gatifloxacin from a single study without data on drug exposure [10, 25]. It was not acknowledged that even high-dose gatifloxacin did not always overcome the low-level MIC increases conferred by *gyrA* A90V and similar mutations [10, 25]. Subsequent PK/PD modelling suggested that this second breakpoint might be clinically useful, but also reinforced the idea that low-level fluoroquinolone resistance is unlikely to be overcome by high-dose moxifloxacin in all patients because of patient-to-patient variability in the moxifloxacin exposure [26]. Nevertheless, given the potentially significant clinical value of using high-dose moxifloxacin when few other treatment options remain, this question should be prioritised for future review using additional data, including the recent studies using the hollow fibre infection model, to provide a more comprehensive and nuanced recommendation to clinicians [27–29]. Similarly, WHO has already announced that it would revisit the rifampicin breakpoint, should a higher dose of rifampicin be endorsed [13].

Considering this complex history, regulators and developers of diagnostics and drugs should fully embrace modern microbiological principles to define breakpoints and associated dosing regimens (figure 1) [17, 30, 31]. To this end, the two systematic reviews provide unprecedented detail about the underlying reasons and scientific evidence for all new recommendations by WHO, to facilitate external scrutiny and to encourage more research where the available evidence was limited [10, 13]. Where possible, these efforts should be coordinated between major regulators to minimise the burden to developers of drugs and AST devices (*e.g.* by recognising a single reference method against which all commercial AST methods are validated) [9]. It would also be beneficial if common AST terminology were adopted to avoid confusion. For instance, the meaning of “clinical breakpoint” differs between regulators (figure 1) and adopting the “area of technical uncertainty” for TB needs further consideration (figure 2) [10, 13]. Regulators should also review if the use of surrogate drugs can minimise false-susceptible results to provide clarity for assay developers about which agents to invest in (*e.g.* whether levofloxacin should be tested as the representative fluoroquinolone and whether kanamycin should be used as a surrogate for amikacin resistance (figure 2)) [22]. Although there has been a great deal of progress in the past 5 years, proactive action by the entire TB community is required to develop an updated AST framework given that the In Vitro Diagnostic Medical Device Regulation will come into effect in the European Union in May 2022. Because previous diagnostic approvals will not be automatically recognised, industry will have to invest to keep its AST devices on the market. We have an obligation to those infected and affected by TB to learn from past experiences and to make the most of this unique window of opportunity [7, 18, 32].

## Shareable PDF

10.1183/13993003.00166-2022.Shareable1 This one-page PDF can be shared freely online.
Shareable PDF ERJ-00166-2022.Shareable
