# Supplementary material for: Updating the approaches to define susceptibility and resistance to anti-tuberculosis agents: implications for diagnosis and treatment
Source: Eur Respir J. 2022 Apr 14;59(4):2200166. doi: 10.1183/13993003.00166-2022 (PMC9059840; doi:10.1183/13993003.00166-2022)
Supplement: Supplementary file 1 [file ERJ-00166-2022.Shareable.pdf]

EUROPEAN RESPIRATORY JOURNAL
EDITORIAL
ANTIMYCOBACTERIAL SUSCEPTIBILITY TESTING GROUP

SHAREABLE PDF

Updating the approaches to define susceptibility and resistance to anti-tuberculosis agents: implications for diagnosis and treatment

Antimycobacterial Susceptibility Testing Group

Collaborators are listed at the end of the article

Corresponding author: Claudio U. Köser (cuk21@cam.ac.uk)

Check for updates

Shareable abstract (@ERSpublications)
Inappropriately high breakpoints have resulted in systematic false-susceptible AST results to anti-TB drugs. MIC, PK/PD and clinical outcome data should be combined when setting breakpoints to minimise the emergence and spread of antimicrobial resistance. https://bit.ly/3i43wb6

Cite this article as: Antimycobacterial Susceptibility Testing Group. Updating the approaches to define susceptibility and resistance to anti-tuberculosis agents: implications for diagnosis and treatment. *Eur Respir J* 2022; 59: 2200166 [DOI: 10.1183/13993003.00166-2022].

This single-page version can be shared freely online.

Copyright ©The authors 2022.

This version is distributed under the terms of the Creative Commons Attribution Licence 4.0.

Received: 24 Jan 2022
Accepted: 5 March 2022

Approximately 85 000 deaths globally in 2019 were due to drug-resistant tuberculosis (TB), which corresponds to 7% of global deaths attributable to bacterial antimicrobial resistance [1]. Yet concerns have been mounting that drug-resistant TB was being underestimated because the approaches to define susceptibility and resistance to anti-TB agents had not kept up with those used for other major bacterial pathogens [2–9]. Here, we outline the recent, evidence-based initiatives spearheaded by the World Health Organization (WHO) and others to update breakpoints (traditionally referred to as critical concentrations (CCs)) that are used for phenotypic antimicrobial susceptibility testing (AST), also called drug susceptibility testing in the TB literature.
